# A novel growth factor bioavailability-enhanced allograft for use in craniomaxillofacial bone regeneration

**DOI:** 10.3389/fbioe.2026.1841387

**Published:** 2026-09-08

**Authors:** Sean A. F. Peel, Jeff Terryberry, Shiva Naseri, Charles F. Orth

**Affiliations:** 1 Red Rock Regeneration Inc., Etobicoke, ON, Canada; 2 Faculty of Dentistry, University of Toronto, Toronto, ON, Canada; 3 Private Practice, Dallas, TX, United States

**Keywords:** bone graft substitutes, bone regeneration, growth factor bioavailability-enhanced allograft, osteoinduction

## Abstract

**Introduction:**

Regeneration of bone in challenging clinical environments requires use of a bone graft substitute that is both osteoregenerative and osteoconductive. Several bone grafts claim osteoregenerative potential, including allogeneic protein-enhanced (OsteoAMP and OsteoFactor), cell-binding peptide-enhanced (i-Factor), recombinant bone morphogenetic protein 2 (BMP-2) containing (INFUSE), or cellular allografts (ViviGen and Trinity). Recently, a novel growth factor bioavailability-enhanced allograft (Natural Matrix Protein®, NMP), was developed that similarly claims osteoregenerative potential. This article evaluates NMP and compares it to other bone grafts.

**Methods:**

The release of BMP-7 into PBS + Tween over 14 days from demineralized bone matrix (DBM) and NMP prepared from the same donors was measured by ELISA. The osteoinductive potential of NMP mixed with different amounts of inactive DBM was assessed in the athymic rat muscle pouch by microCT and histomorphometry. A second muscle pouch study evaluated the osteoinductive and osteogenic properties of OsteoAMP, OsteoFactor, i-Factor, ViviGen, Trinity, and INFUSE compared to NMP. The clinical potential of NMP to regenerate a severely atrophic maxilla and support dental implant placement was evaluated in two cases.

**Results:**

NMP released significantly more BMP-7 than DBM over 14 days. NMP-iDBM mixtures were osteoinductive when the mixture contained 100%, 75%, and 50% NMP. MicroCT analysis of OsteoAMP, i-Factor, ViviGen, and Trinity showed they all contained mineralized tissue prior to implantation, confounding microCT evaluation of the recovered implants. Histological analysis showed that OsteoAMP, OsteoFactor, and i-Factor failed to form bone *in vivo*. The cellular allografts produced new bone inconsistently, while INFUSE and NMP consistently formed bone. The clinical cases showed that the use of NMP resulted in bone regeneration of sufficient volume and quality to support placement and function of dental implants.

**Conclusion:**

NMP is osteoinductive *in vivo* and can support bone regeneration clinically.

## 1 Introduction

Achieving bone regeneration clinically can be a significant challenge depending on the degree of trauma at the site, size of the defect, lack of vascularity, underlying comorbidities (age, diabetes, smoking, infection, and prior bone loss), and the effects of concomitant medications including bisphosphonates and cancer therapeutics, resulting in the requirement to use bone grafts to enhance the body’s natural osteoregenerative potential (Marsh and Li, 1999; Ferraz, 2023). One of the more challenging situations in craniomaxillofacial surgery is restoration of the atrophic maxilla to support successful dental implant placement, which requires that the regenerated bone of the restored ridge is of sufficient height, width, and quality to support loading of the implants (Aghaloo et al., 2016).

Autogenous bone graft (ABG) comprising cancellous bone and marrow (i.e., harvested from the iliac crest) is considered the gold standard bone graft material, as it provides a scaffold, osteogenic cells, and growth factors. However, there are well-recognized problems associated with ABG, including increased operating time, blood loss, donor site infection, and pain (Arrington et al., 1996). This has led to the use of a vast array of different bone graft substitutes, including freeze-dried bone allograft (FDBA), demineralized bone matrix (DBM), allogeneic protein-enhanced grafts, cellular bone allografts (CBAs), xenografts, peptide-enhanced xenografts, synthetic grafts, and recombinant growth factor-containing grafts (Ferraz, 2023; Laurencin et al., 2014).

Bone graft substitutes can be categorized by the proposed mechanisms by which they support bone regeneration, including osteoconduction, where they act as a scaffold for tissue ingrowth, osteogenesis, where they contribute bone-forming cells, and osteoinduction, where they provide growth factors (BMPs) that induce host cells to differentiate into bone-forming cells (Albrektsson and Johansson, 2001). In addition, some grafts are osteopromotive as they provide additional biological signals to enhance vascularization or mesenchymal cell recruitment (Dimar et al., 2007).

Recently, a novel growth factor bioavailability-enhanced allograft called Natural Matrix Protein® (NMP), has been developed. It was shown to have increased release of osteoinductive growth factors *in vitro* and osteoinductive activity *in vivo* compared to DBM and formed more bone than the recombinant BMP-2-containing graft INFUSE, even though it contained less BMP (Smith et al., 2026).

This article compares the osteoregenerative potential of NMP to various other bone graft substitutes across the different categories in a muscle pouch model that allows for evaluating osteoinductive and osteogenic potential and then reports on two clinical cases in which NMP was used to restore atrophic maxillae prior to dental implant placement.

## 2 Materials and methods

### 2.1 Assessment of BMP bioavailability *in vitro*

Cortical bone samples from three research-consenting human donors were separately ground into microparticulates (0.25–1 mm in diameter) and then processed into demineralized bone matrix (DBM) or NMP as previously described by Smith et al. (2026).

Triplicate 100 mg preparation replicates of each test article were placed in sterile 15 mL centrifuge tubes, and 8 mL of PBS + 0.05% Tween 20 + 0.05% sodium azide (PBST) were added to each tube. The tubes were placed in an incubator at 37 °C and rocked on a nutator. On days 1, 3, 7, and 14, the tubes were centrifuged, and the supernatant was removed and frozen for analysis. On days 1, 3, and 7, the solution was replaced with fresh PBST. On day 14, the solution was replaced with 4 M guanidine HCl 10 mM Tris pH 7.4 + 0.05% Tween 20 (GDNT), and the matrix was incubated at room temperature with rocking for a further 24 h. Following this, the GDNT extract was recovered, and buffer exchanged into ELISA diluent buffer using 10 kDa MWCO Amicon filters (Millipore Sigma, St Louis, MO). The samples were frozen until they were ready for ELISA analysis. The BMP-7 in each extract was measured using a BMP-7 Quantikine ELISA Kit (R&D Systems, Minneapolis, MN) according to the manufacturer’s instructions.

The amount of BMP-7 in each extract was normalized to the dry weight of the test article, and the cumulative amount of BMP released at each time point was calculated as a percentage of the total amount of BMP-7 extracted. The percentage of BMP-7 released into PBST from DBM and NMP for the three donors was compared using multiple linear regression (GraphPad Prism 10, version 10.3.1; GraphPad Software, Boston, MA).

### 2.2 Correlation of microCT to histomorphometry when evaluating bone formation in the athymic rat muscle pouch model

The correlation between microCT and histomorphometric evaluation of NMP-induced osteoinduction was assessed in the muscle pouch model. Surgeries were performed by IBEX Preclinical Research (Logan, UT, United States) in accordance with ASTM F2529-13 (ASTM, 2013) under the Institutional Animal Care and Use Committee (IACUC) Protocol: IBEX IACUC IBEX 23-06.

Bovine demineralized bone matrix was inactivated by incubation with 4 M guanidine HCl, followed by water rinses, incubation with 0.13 M acetic acid, neutralization with sodium phosphate, and water rinses. The inactive DBM (iDBM) was lyophilized and stored at 2–8 °C. Bovine NMP was prepared as described above. The lyophilized NMP granules were mixed with the iDBM granules to prepare a series of test articles with 100%, 75%, 50%, 25%, or 0% NMP (w/w). These were then sterilized by gamma irradiation.

A total of 25 male athymic nude rats (rnu−/rnu−), approximately 7 weeks old, were divided into five groups of five animals, and two implants were placed per animal (n = 10 per group). At the time of surgery, the test articles were rehydrated with sterile saline (2.0 mL/2.5 cc), and 0.2 cc was placed in an intermuscular pocket between the semimembranosus and semitendinosus muscles in each rear limb.

Implants were recovered after 28 days, fixed in 10% formalin, and scanned by microCT (SKYSCAN 1276 Bruker Preclinical Imaging, Burlington, Canada). The recovered explanted tissue was then demineralized, embedded in paraffin, sectioned along the long axis at the center of the recovered implant, and stained with hematoxylin and eosin (H&E).

MicroCT analysis was performed by a blinded reviewer using Analyze 15.0 (Analyze Direct, Stilwell, KS) as described by Smith et al. (2026). In brief, the implant was outlined as a region of interest (ROI) and was segmented into “non-bone” (voxels above background density but less than bone) and “bone” (voxels >100 mg/cc). Mean intensity and volume were measured for each object individually and in combination. Percent mineralized tissue (%MIN), a measure of the quality of new bone, was calculated by dividing bone volume by total implant volume and multiplying by 100.

Histologic osteoinductivity (OI) scores were determined following ASTM F2529-13 (ASTM, 2013). In brief, slides were digitally scanned, and image files were analyzed using ZEISS ZEN 3.11 imaging software. Three sections were assessed for each explant. The largest visible cross-section was selected, and a 600 × 600 µm grid was overlaid on each section. A blinded reviewer assessed each square, assigning a score of 0 or 1. Squares with a score of 1 contained bone-forming elements, including new bone, osteoblasts, osteocytes, cement lines, bone marrow, or cartilage. Percent new bone (%NB) was calculated as the number of positive squares divided by the total number of squares scored. Final OI scores were as follows: 0 = no implant detected; 1 = <10% NB; 2 = 10–20% NB; 3 = 21–30% NB; and 4 = >30% NB. Typically, 400+ squares per section were counted.

### 2.3 Evaluation of osteoinductive and osteogenic potential of various bone graft substitutes in the athymic rat muscle pouch model

A second muscle pouch study was performed to assess the osteoinductive and osteogenic potential of various bone graft materials representing various categories. The materials were evaluated in the muscle pouch model at IBEX under IACUC Protocol: IBEX IACUC 20–10 Amendment 31.

A total of 35 male athymic nude rats (rnu−/rnu−), approximately 8 weeks old, were divided into seven groups of five animals each, and two implants were placed per animal (n = 10 per group, with the NMP group being from two different lots). All test articles were purchased from their manufacturers and were stored and prepared as described in their instructions for use (IFUs) (Table 1). Additional information on the manufacturer and product description is provided in Supplementary Table S1.

**TABLE 1:** Storage and preparation of test articles.

Test article	Form	Storage temperature	Preparation
OsteoAMP	Granules	11–25 °C	Hydrated with lactated Ringer’s solution (LRS) for 10 min. A 0.2 cc volume was aseptically loaded into each syringe
OsteoFactor	Vial of powder	11–30 °C	Hydrated with 3.0 cc sterile saline, then added to unhydrated OsteoAMP and incubated for 15 min. A 0.2 cc volume was aseptically loaded into each syringe.
i-Factor	Putty	Ambient	Expelled into a sterile aluminum weigh boat. A 0.2 cc volume was aseptically loaded into each syringe
ViviGen	DBM fibers combined with cancellous chips with viable cells in a cryoprotectant	<-70 °C	Transported from freezer and placed in its packaging into a 37 °C water bath within 8 min. After thawing, discharged into a sterile aluminum weigh boat. A 0.2 cc volume was aseptically loaded into each syringe
Trinity elite	DBM fibers combined with cancellous chips with viable cells in a cryoprotectant	−70 °C to −80 °C	Thawed in its packaging in a 37 °C water bath. Sterile gauze was used to decant the cryopreservation solution. The article was hydrated with 5% dextrose in LRS (D5LR) to the fill line. D5LR was removed prior to loading a 0.2 cc volume aseptically into each syringe
INFUSE	Vial BMP powder, vial water, ACS	15–30 °C	Lyophilized rhBMP-2 was reconstituted with sterile water, and 0.7 mL of the resulting 1.5 mg/mL BMP solution was applied to the ½ × “2”(∼2 cc) collagen sponge for a minimum of 15 min. The sponge was then cut into 10 pieces, each containing 0.105 mg rhBMP-2. Sponges were directly implanted.
NMP	Granules	Ambient	Hydrated with saline for a minimum of 10 min and then a 0.2 cc volume was aseptically loaded into each syringe Five implants were from one lot and five implants were from a second lot (different donors)

Except for INFUSE, following preparation of the test articles, 0.2 cc implants of each were loaded into open-ended 1 mL syringes and then placed into the muscle pouch that had been formed in both hind limbs by blunt dissection. For INFUSE, the collagen sponge (∼2 cc) was rehydrated with 0.7 mL of BMP solution (1.5 mg/mL) and allowed to incubate for 20 min, after which it was cut into 10 pieces containing ∼105 µg rhBMP-2. The pieces were placed directly into the muscle pouch without use of the syringe to prevent expressing BMP-2 solution from the sponge.

Implants were recovered after 28 days, fixed in 10% formalin, scanned by microCT (Quantum FX, PerkinElmer, MA), demineralized, embedded in paraffin, sectioned along the long axis at the center of the recovered implant, and stained with hematoxylin and eosin (H&E).

For each test article except INFUSE, the residual unimplanted test article was fixed immediately and stored until it was scanned by microCT, demineralized, and processed for histological evaluation along with the recovered implants.

The %MIN in each recovered explant was determined by microCT. Incidence of bone formation was assessed by histological review of three sections from each recovered implant. When bone-forming elements were identified within the slides, the implant was scored 1; otherwise, it was scored 0.

### 2.4 Case studies

This case study was approved by the Veritas Central Institutional Review Board (IRB) with a consent waiver (2026-3932-23825-2).

#### 2.4.1 CASE 1: horizontal and vertical deficiency of the left anterior maxilla

**FIGURE 1:**
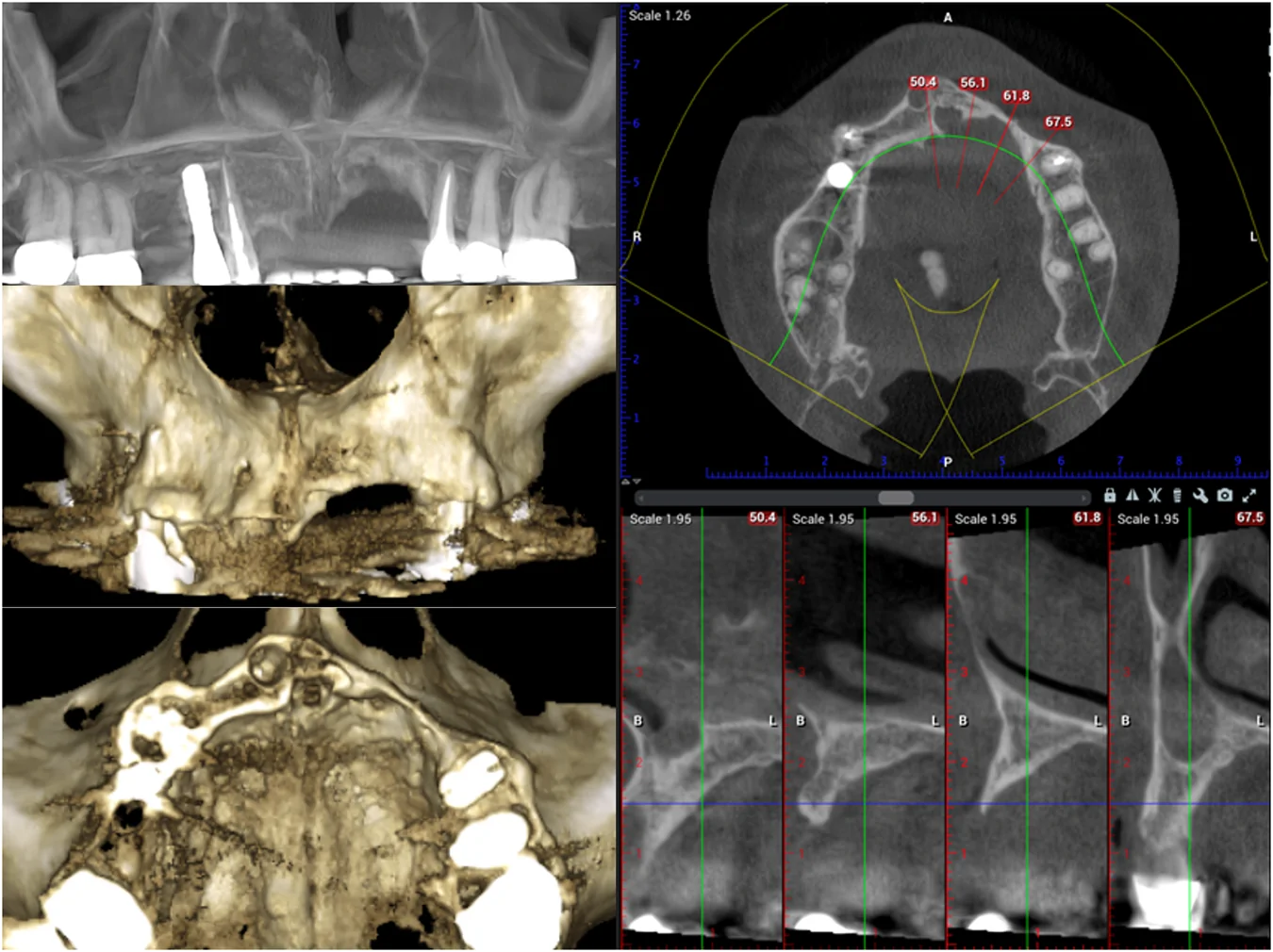
Case 1 Pre-op. Cone beam CT (CBCT) images show an area of extensive loss of bone height on the reconstructed panoramic radiograph and loss of bone width in the axial plane and cross-sectional slices. Top left: Panoramic radiograph. Middle left and bottom left: 3D reconstruction. Top right: CBCT axial view. Bottom right: CBCT coronal view. The numbers in the axial view represent angular positions of the gantry relative to the patient and match the coronal sections.

The subject was a healthy 73-year-old female with a history of failed maxillary implants more than 10 years ago who had been wearing a partial anterior maxillary prosthesis since that time. Recently, her maxillary right canine tooth fractured, making the denture uncomfortable to wear. The patient desired to have implants replaced in the left anterior maxilla so that she could return to functioning with an implant-supported restoration. The patient’s preoperative cone beam CT scans demonstrated horizontal and vertical alveolar ridge deficiency in the left anterior maxilla (Figure 1).

The deficient anterior maxilla was augmented with 2.5 cc of NMP Fibers (Induce Biologics USA, Irvine, CA) rehydrated with blood. A perforated polytetrafluoroethylene (PTFE) titanium membrane (Osteogenics, Lubbock, TX) was then placed over the fiber bioimplant to maintain ridge shape and prevent compression of the graft. A perforated membrane was selected to allow for cellular ingrowth into the graft.

#### 2.4.2 Case 2: horizontal and vertical deficiency of the left anterior maxilla

**FIGURE 2:**
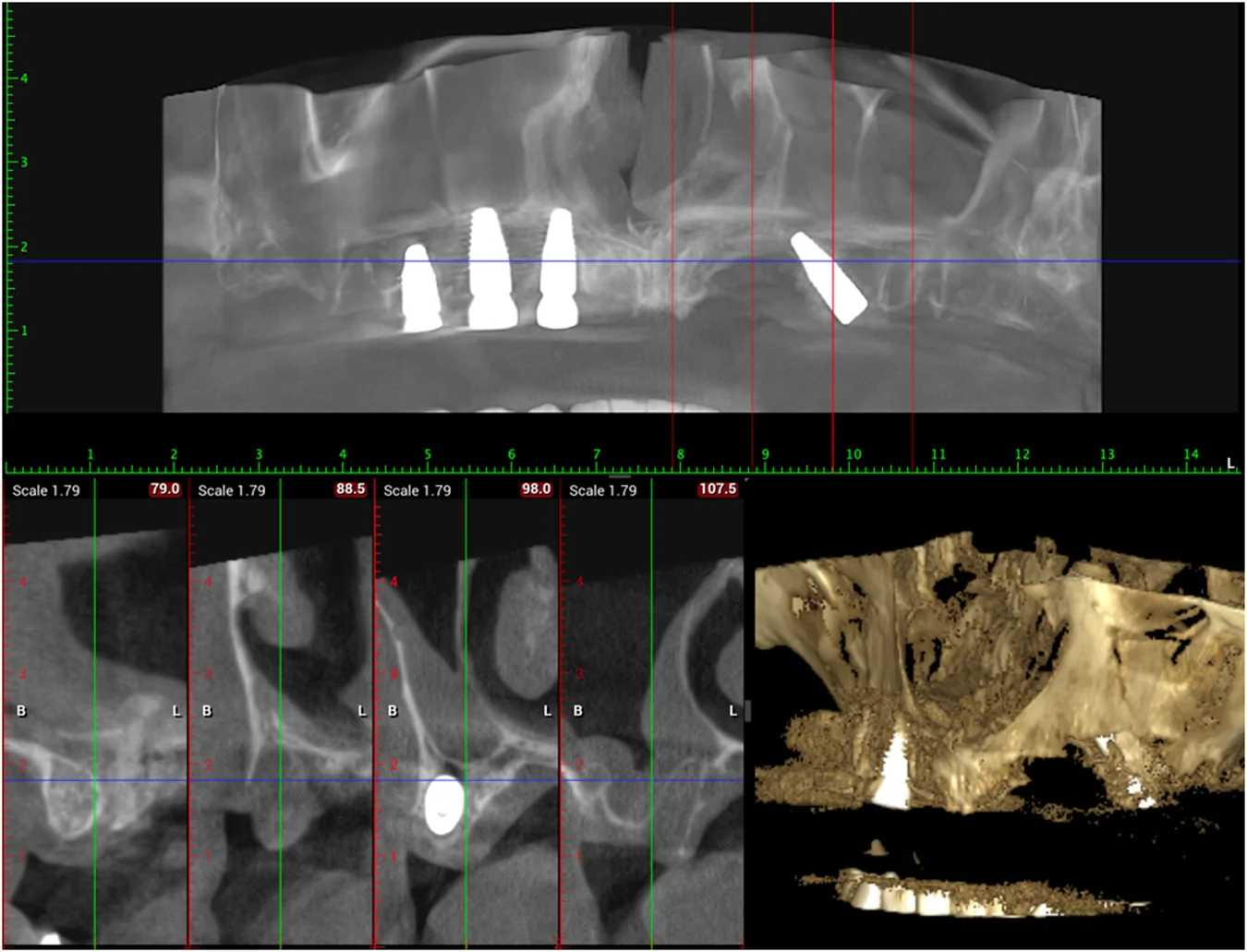
Case 2 Pre-op. Pre-op CBCT shows a 3 cm region with significant loss in bone height in the reconstructed panoramic radiograph (top) and loss of bone width in the sagittal slices (bottom left) and 3D reconstruction (bottom right).

The subject was a 28-year-old Caucasian male with a history of long-term drug abuse with no other medically related issues. The patient was initially treated to remove his existing maxillary teeth and lower molars and restore dental caries on the premolars and incisors. Once healed, six dental implants were placed. Unfortunately, the patient experienced bone loss during the healing phase of the upper left implants. Those implants needed to be removed because of bone loss and inadequate support (Figure 2).

**FIGURE 3:**
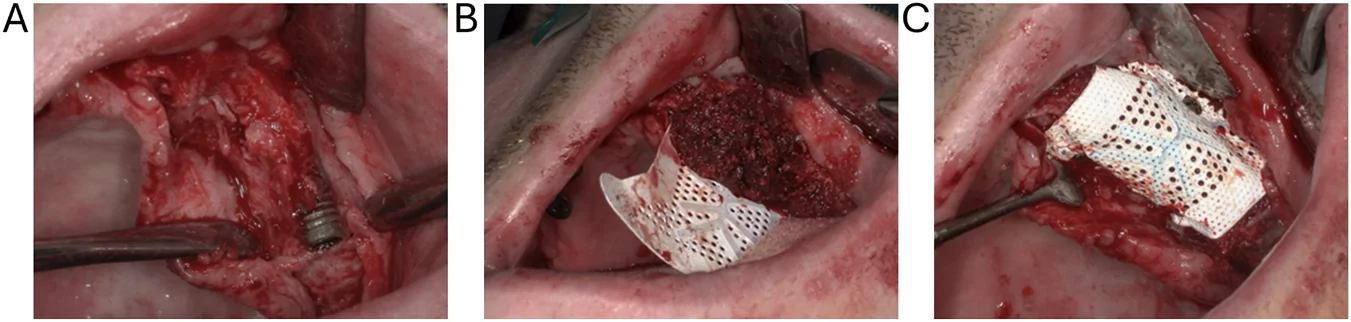
Case 2 Intra-op. Intra-operative photographs showing removal of failed implant **(A)**, placement of rehydrated NMP graft **(B)**, and placement of the membrane **(C)**.

The decision was made to augment the deficient anterior maxilla with 2.5 cc of NMP fibers rehydrated with blood prior to implant placement. Following placement of the fiber bioimplant, a perforated PTFE titanium membrane was placed over the graft (Figure 3).

## 3 Results

### 3.1 Assessment of BMP bioavailability *in vitro* (Figure 4)

**FIGURE 4:**
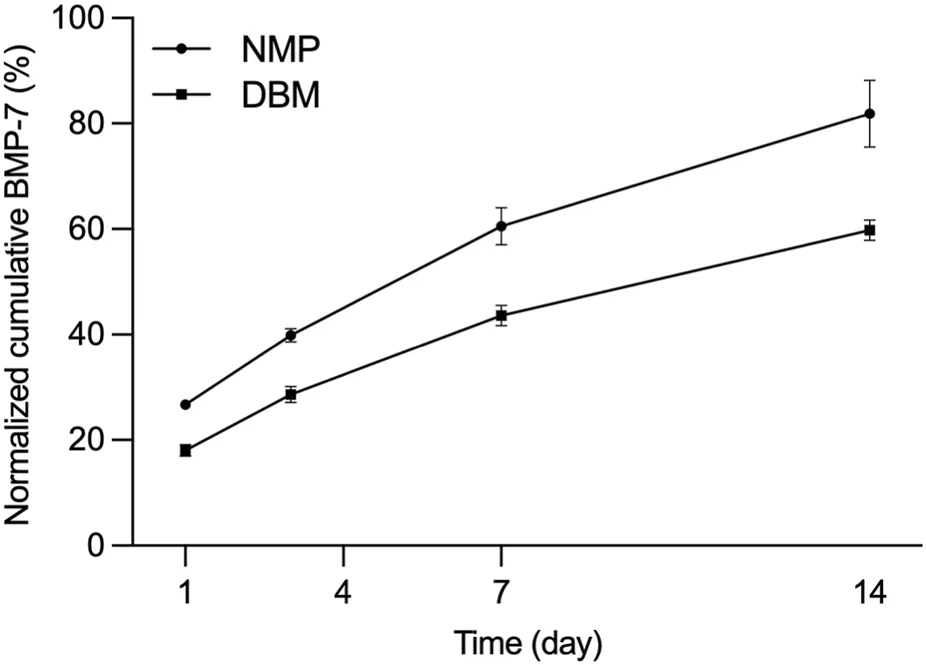
Release of BMP-7 from DBM and NMP. NMP and matching DBM produced from three human donors (mean ± SEM of the 3 donors, n = 3 replicates per donor) were incubated with PBS + Tween (PBST) for 14 days at 38 °C. Significantly more BMP-7 was released from the NMP than DBM (*p* = 0.009).

The BMP-7 released from NMP on days 1, 3, 7, and 14 was approximately 40% higher than that released from DBM at the same time points (Figure 4). Statistically, both time and matrix (NMP vs. DBM) had a significant effect on the amount of BMP-7 released, with *p*-values of 0.0002 for time and 0.009 for matrix.

### 3.2 Correlation of microCT to histomorphometry (Table 2)

Both the %MIN measured by microCT and %NB measured histologically did not decline between the 100% NMP and the 75% NMP groups. However, they both showed a dose-dependent decline from 75% to 0% NMP (Table 2). The %MIN was highly correlated to the %NB with the coefficient of determination (R^2^) = 0.97 using the least-squares regression method for a linear trendline.

**TABLE 2:** Correlation of microCT to histomorphometric analysis of osteoinduction.

NMP/iDBM	TV (mm^3^) (mCT)	%MIN (mCT)	%NB (histomorphometry)	# implants with NB
100/0	136 ± 41	31.0 ± 6.4	43.1 ± 10.6	10/10
75/25	135 ± 97	35.3 ± 12.9	43.6 ± 12.1	10/10
50/50	125 ± 36	22.5 ± 10.1	30.8 ± 16.5	10/10
25/75	118 ± 44	11.0 ± 8.9	8.9 ± 6.4	8/10
0/100	96 ± 76	5.1 ± 4.2	5.3 ± 3.8	8/9

NMP was mixed with inactive DBM to produce five groups (n = 10 implants per group), which were implanted in the muscle pouch of athymic rats. At 28 days, the implants were recovered, fixed, and analyzed by microCT to measure total volume (TV). The percent volume containing mineralized tissue (%MIN) was analyzed by histomorphometry to estimate the amount of new bone-forming elements (%NB), which includes bone, cartilage, and marrow. The %MIN highly correlated with %NB (R^2^ = 0.97).

### 3.3 Comparison of osteoinductive and osteogenic potential to other bone grafts *in vivo*

#### 3.3.1 MicroCT analysis of unimplanted test articles (Figure 5)

**FIGURE 5:**
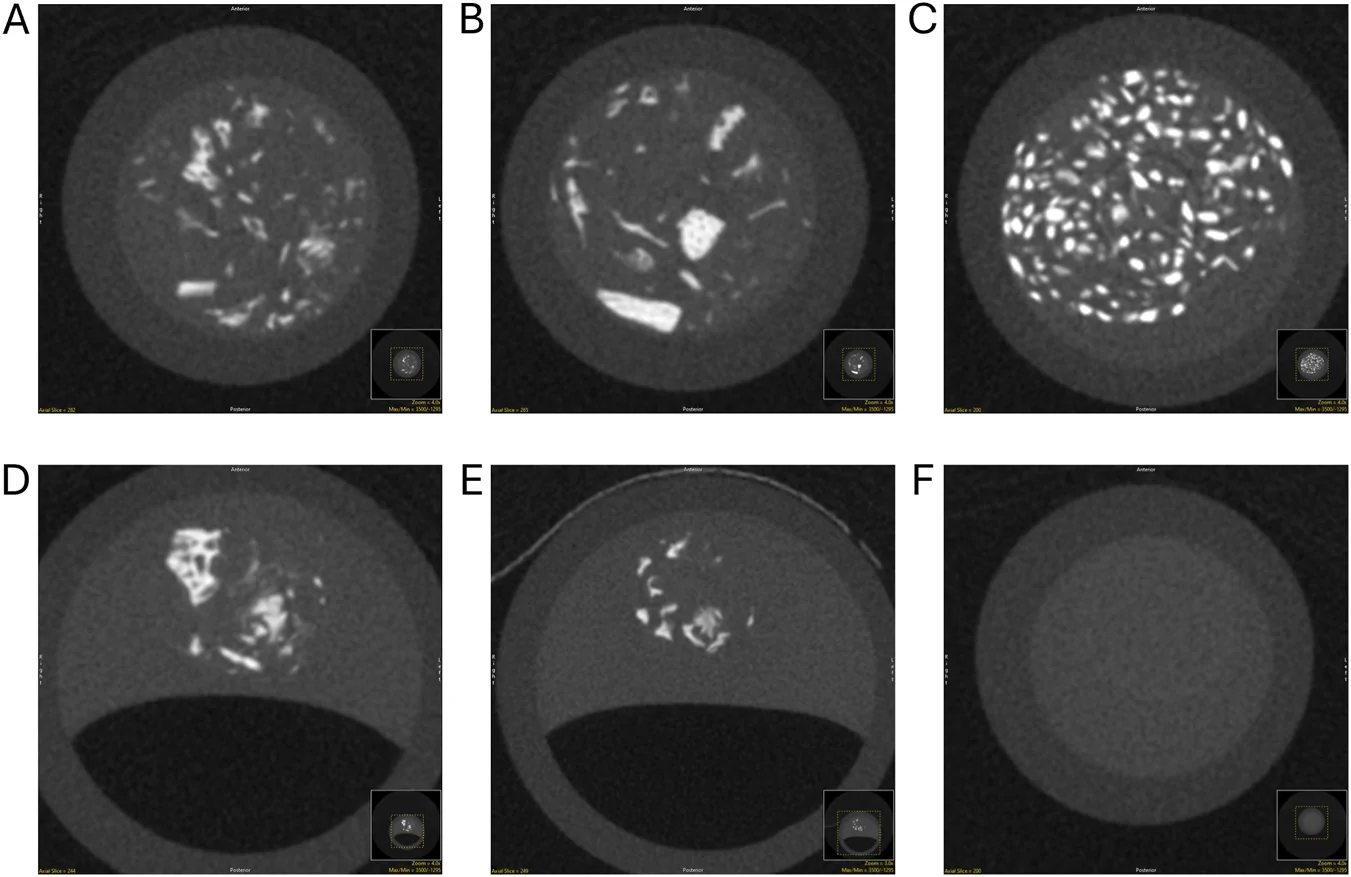
MicroCT of unimplanted test articles. The slice views showing the presence of mineralized tissue in the OsteoAMP **(A)**, OsteoFactor mixed with OsteoAMP **(B)**, i-Factor **(C)**, ViviGen **(D)**, and Trinity **(E)** grafts. In contrast, the NMP graft contained no mineralized tissue prior to implantation **(F)**. Original magnification ×4.

The unimplanted OsteoAMP, OsteoFactor (combined with OsteoAMP), i-Factor, ViviGen, and Trinity all contained mineralized tissue (Figures 5A–E). No mineralized tissue was present in the unimplanted NMP (Figure 5F). As all the INFUSE was implanted, no unimplanted INFUSE was available for assessment.

#### 3.3.2 MicroCT of recovered implants (Figure 6; Table 3)

**FIGURE 6:**
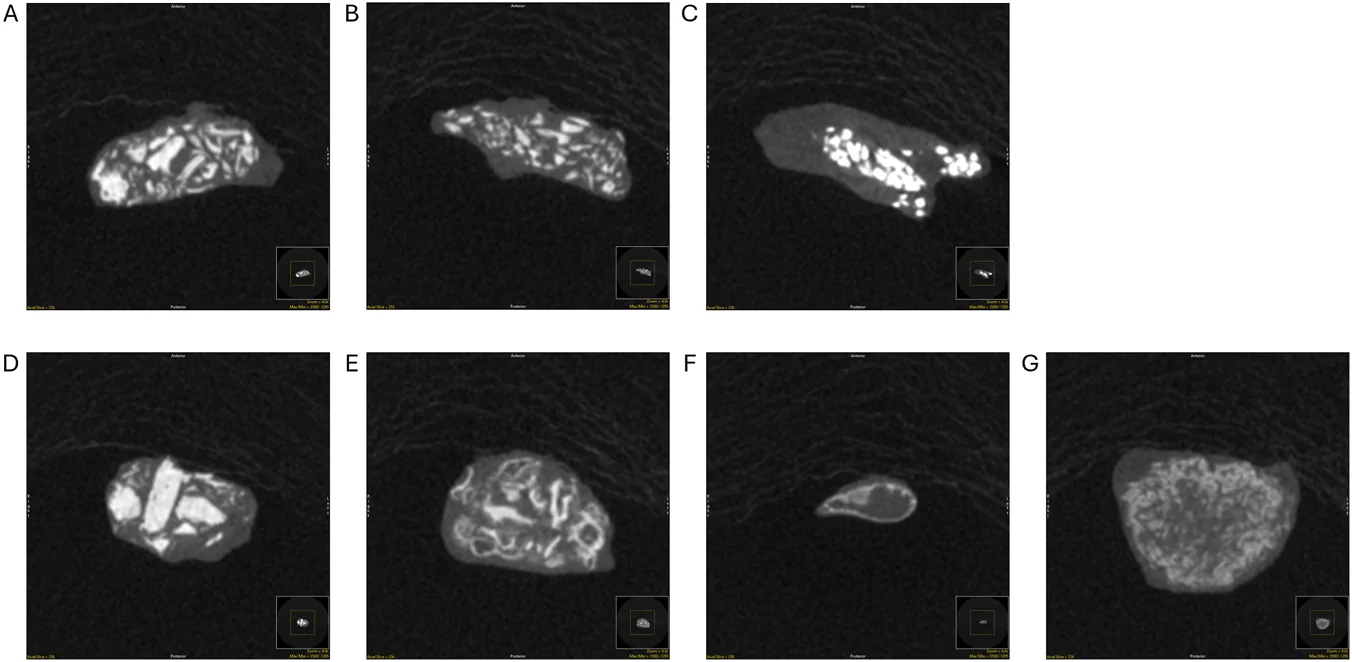
MicroCT of recovered implants at 28 days. It was not possible to identify the presence of new bone in the OsteoAMP **(A)**, OsteoFactor mixed with OsteoAMP **(B)**, i-Factor **(C)**, ViviGen **(D)**, and Trinity **(E)** grafts due to the pre-existing mineralized tissue present within these grafts. The INFUSE graft showed the formation of a thin shell of mineralized tissue around a nonmineralized core **(F)**, while the NMP graft showed newly formed mineralized tissue throughout the implant **(G)**. Original magnification ×4.

MicroCT imaging of the OsteoAMP and the OsteoFactor + OsteoAMP recovered implants appeared similar to the unimplanted samples, with large irregular, well-defined chips of highly mineralized tissue observed throughout each implant. Some smaller fiber-like material was also observed (Figures 6A,B).

**TABLE 3:** MicroCT and histological evaluation of recovered implants.

Test article	Unimplanted %MIN (mCT)	Implanted TV (mm^3^) (mCT)	Implanted %MIN (mCT)	# Implants with NB
OsteoAMP	12.5%	216 ± 26	44.8 ± 5.5%	0/10
OsteoFactor + OsteoAMP	10.6%	228 ± 47	40.9 ± 8.1%	0/10
i-Factor	18.2%	199 ± 54	17.9 ± 15.4%	0/10
Vivigen	10.5%	291 ± 57	43.7 ± 6.7%	7/10
Trinity	3.4%	239 ± 36	38.4 ± 5.3%	10/10
INFUSE	ND	29 ± 13	30.8 ± 10.2%	10/10
NMP-1	0.0%	59 ± 33	17.2 ± 9.5%	5/5
NMP-2	0.0%	120 ± 57	30.3 ± 8.1%	5/5

Various test articles (n = 10 implants per group) were implanted in the muscle pouch of athymic rats. At 28 days, the implants were recovered, fixed, and analyzed by microCT to measure total volume and the percent volume containing mineralized tissue (%MIN) and by histomorphometry to estimate the amount of new bone-forming elements (%NB), including bone, cartilage, and marrow. The residual unimplanted test articles were also fixed and assessed for %MIN by microCT.

The i-Factor recovered implants also appeared similar to the unimplanted sample, with small, well-defined high-intensity “granules” present throughout the recovered implant (Figure 6C).

ViviGen and Trinity both appeared to have small chips and fibers of high intensity and some thin, fibrous areas of moderate intensity. One or two small ossicle-like structures (moderate intensity mineralized ring, surrounding non-mineralized core) were observed in some implants. These were more common and larger in the Trinity implants (Figures 6D,E) and were suggestive of newly formed bone ossicles.

The INFUSE implants showed a single well-defined ossicle with a moderately intense ring of mineralized tissue surrounding an unmineralized core. These ossicles were significantly smaller than the mineralized masses of the other test articles (Figure 6F).

The recovered NMP implant had a moderate-intensity punctate appearance throughout the recovered implant volume, which was suggestive of bone formation throughout the implant (Figure 6G).

The total volume (TV) and %MIN results for the various groups are summarized in Table 3.

### 3.4 Histological analysis (Figure 7)

Non-vital bone matrix (NVB) was identified based on the absence of osteocytes within the widely spaced lacunae and the matrix appearing lamellar. New bone (NB) was identified based on the presence of cells within lacunae that were irregularly spaced and the matrix appearing woven and staining darker than the NVB. New bone formed on NVB. The marrow had a cobweb appearance with empty spaces surrounded by thin stroma, and it was surrounded by NB. Sinuses and blood vessels containing red blood cells were observed within the marrow.

**FIGURE 7:**
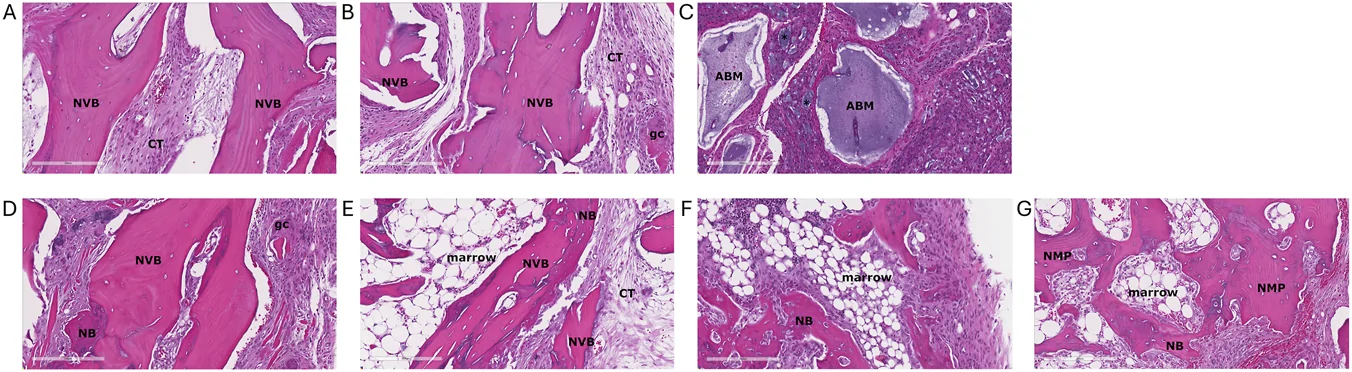
H&E staining of implants 28 days after implantation. No new bone was visible in the OsteoAMP **(A)**, OsteoFactor mixed with OsteoAMP **(B)**, or i-Factor **(C)** implants. Most ViviGen implants (7 of 10) showed islands of NB formation, in contact with NVB **(D)**. All Trinity implants had small ossicles of NB apposed to the NVB associated with bone marrow **(E)**. All the INFUSE implants had a single ossicle comprising a thin shell of NB surrounding bone marrow and remnants of the collagen sponge **(F)**. All the NMP implants had NB apposed to the NMP matrix, which was often associated with marrow **(G)**. Occasional islands of cartilage were also observed. Non-vital bone matrix (NVB). New bone (NB). Anorganic bone matrix (ABM). Cellular infiltrate (CT), giant cells (gc), fragments of ABM (*). Original magnification: ×20; scale bar: 200 µm; field of view for each panel: 850 µm.

OsteoAMP and OsteoFactor + OsteoAMP implants had large non-vital bone chips surrounded by cellular infiltrate (CT). Smaller non-vital bone fragments were also visible. No new bone was observed in any implant. Occasional giant cells (gc) were observed associated with the smaller bone fragments (Figures 7A,B).

i-Factor implants had a dense cellular infiltrate surrounding purple-stained, anorganic bone matrix (ABM) granules. Small fragments of ABM (*) were also observed. No new bone was observed in any implant (Figure 7C).

ViviGen implants comprised large non-vital bone chips with loose stromal tissue within vascular channels in the large chips that were surrounded by a cellular infiltrate. Thin elongated fragments of non-vital bone and bone fragments were also observed throughout the implant (Figure 7D). Seven of the ten implants had areas of new bone (NB). Small ossicles containing marrow were observed in some implants.

Trinity implants comprised non-vital bone chips surrounded by loose cellular infiltrate. One or more small-to-medium-sized ossicles were observed in each of the 10 implants where new bone formed on the allograft chips and surrounded marrow (Figure 7E).

All 10 INFUSE implants comprised a single ossicle with a thin shell of new bone surrounding fatty marrow and residual collagen sponge. These ossicles were significantly smaller than the other implants (Figure 7F).

NMP implants comprised NMP granules associated with cellular infiltrate or fused with newly formed bone throughout the implant (Figure 7G). All 10 implants had ossicles of new bone surrounding bone marrow. Occasional islands of cartilage were also observed. No signs of inflammation or edema were observed associated with the NMP implants.

Representative low-magnification images of the recovered implants from each test article are provided as Supplementary Figure S2.

### 3.5 Case studies

#### 3.5.1 Case 1

**FIGURE 8:**
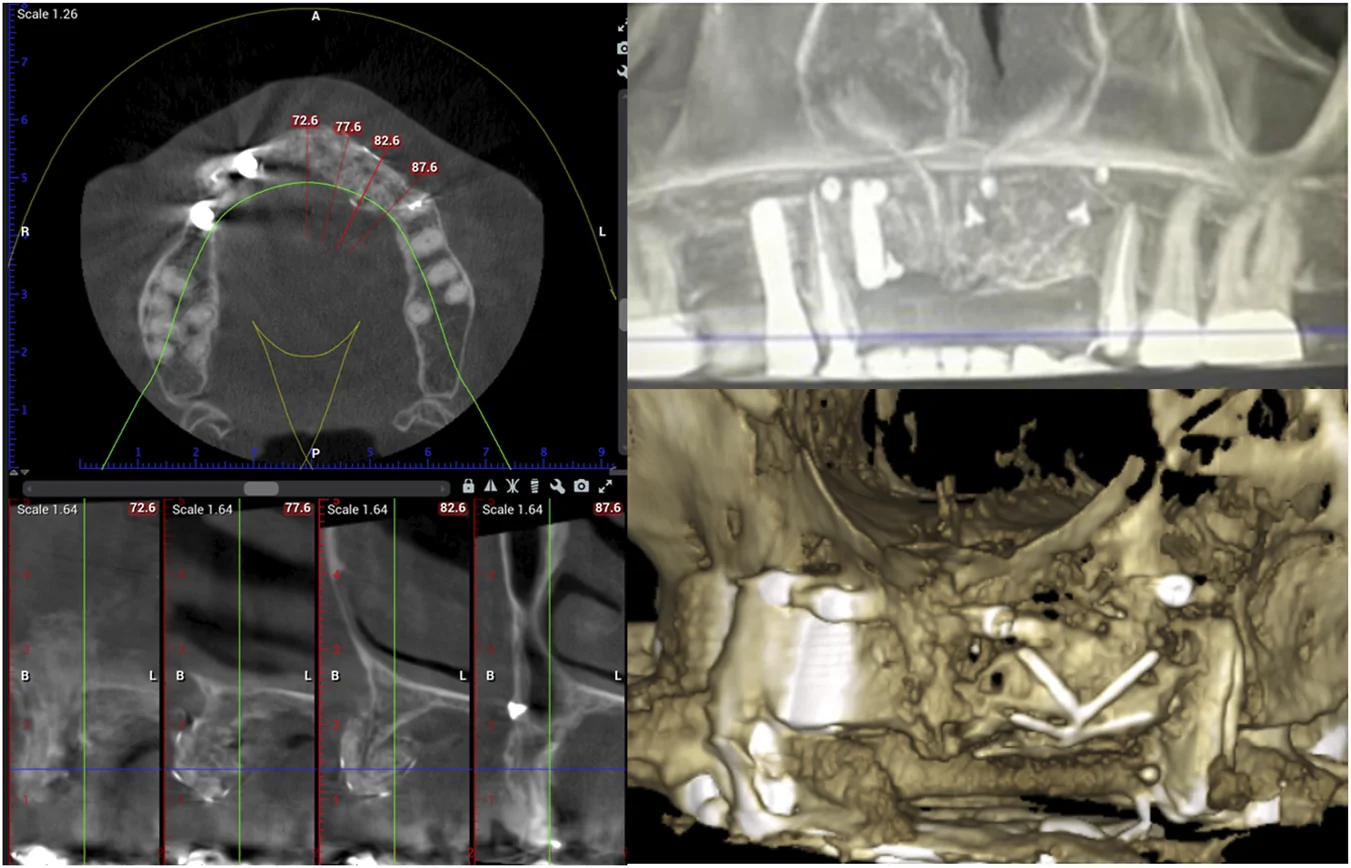
Case 1: 6 months post-grafting. Images show increased bone volume at grafted sites resulting in increased ridge height and width. Top left, CBCT axial view. Bottom left, coronal view. Top right, panoramic radiograph. Bottom right, 3D reconstruction.

**FIGURE 9:**
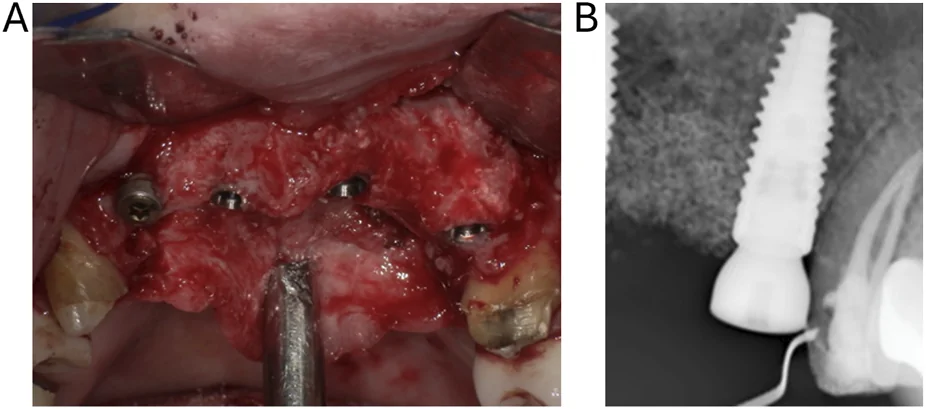
Case 1: 8 months post-grafting. Clinical image **(A)** showing implant placement into good quality bone. Radiograph shows newly regenerated bone along the full length of the implant **(B)**.

Excellent reconstruction of both vertical height and horizontal width was observed at 6 months. Cross-sectional images and 3D imaging demonstrate the desired alveolar ridge form was achieved (Figure 8). At 8 months post grafting, the patient had the alveolus exposed, the membrane removed, and three dental implants (4.1 mm D × 10 mm L) placed into the grafted region. The grafted bone was found to be Type II in density at the time of dental implant insertion (Figure 9).

**FIGURE 10:**
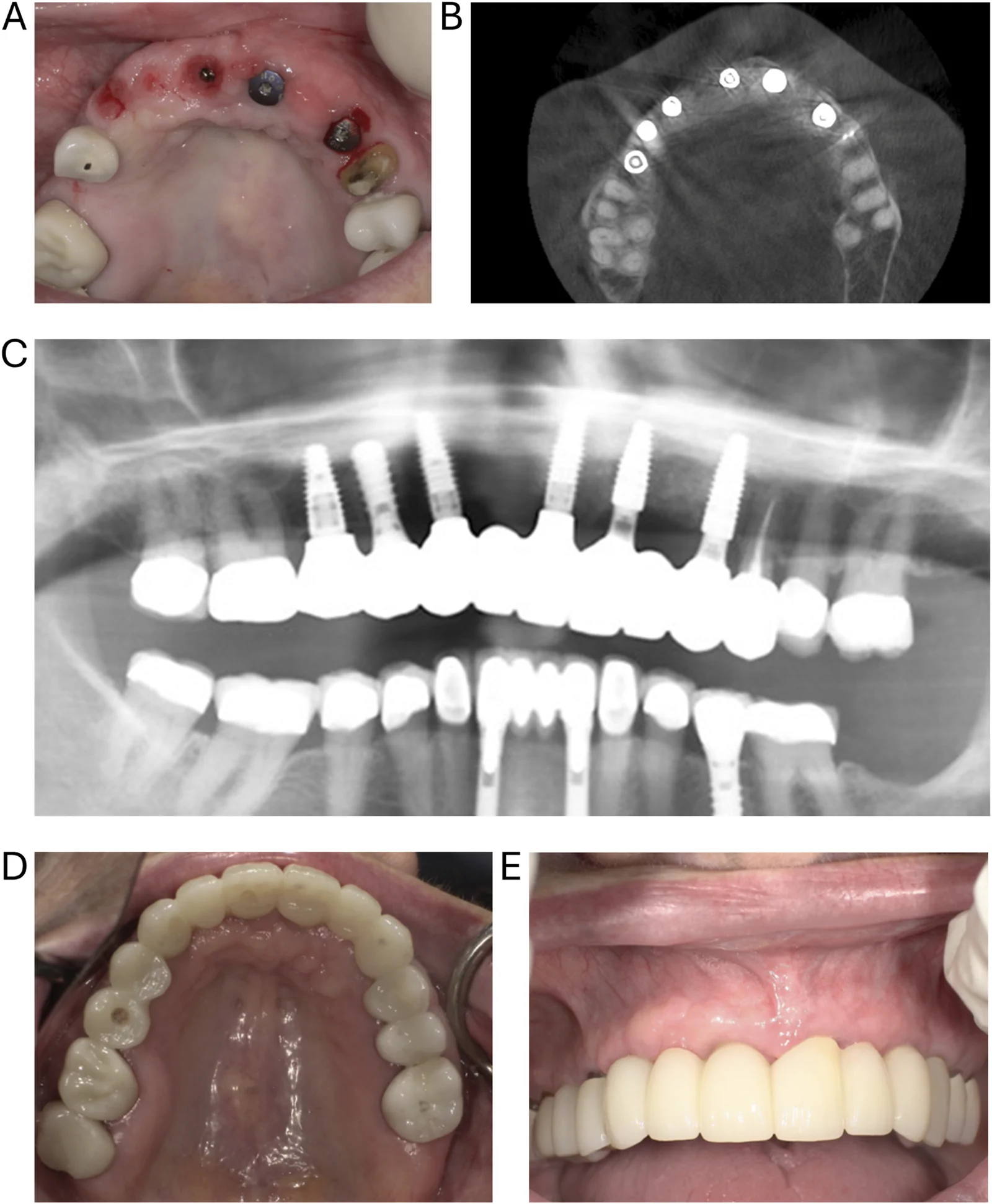
Case 1: post-implant placement. Four months **(A,B)** and six months **(C,D,E)** post-implant placement showing the implants are well integrated, resulting in a good esthetic appearance **(D,E)**.

The radiographic image at 4 months post-implant placement (12 months post grafting) demonstrated integration of the three implant fixtures (Figures 10A,B). At 6 months post-implant placement, the restoration was placed (Figure 10C), resulting in an excellent esthetic and functional prosthetic result (Figures 10D,E).

#### 3.5.2 Case 2

**FIGURE 11:**
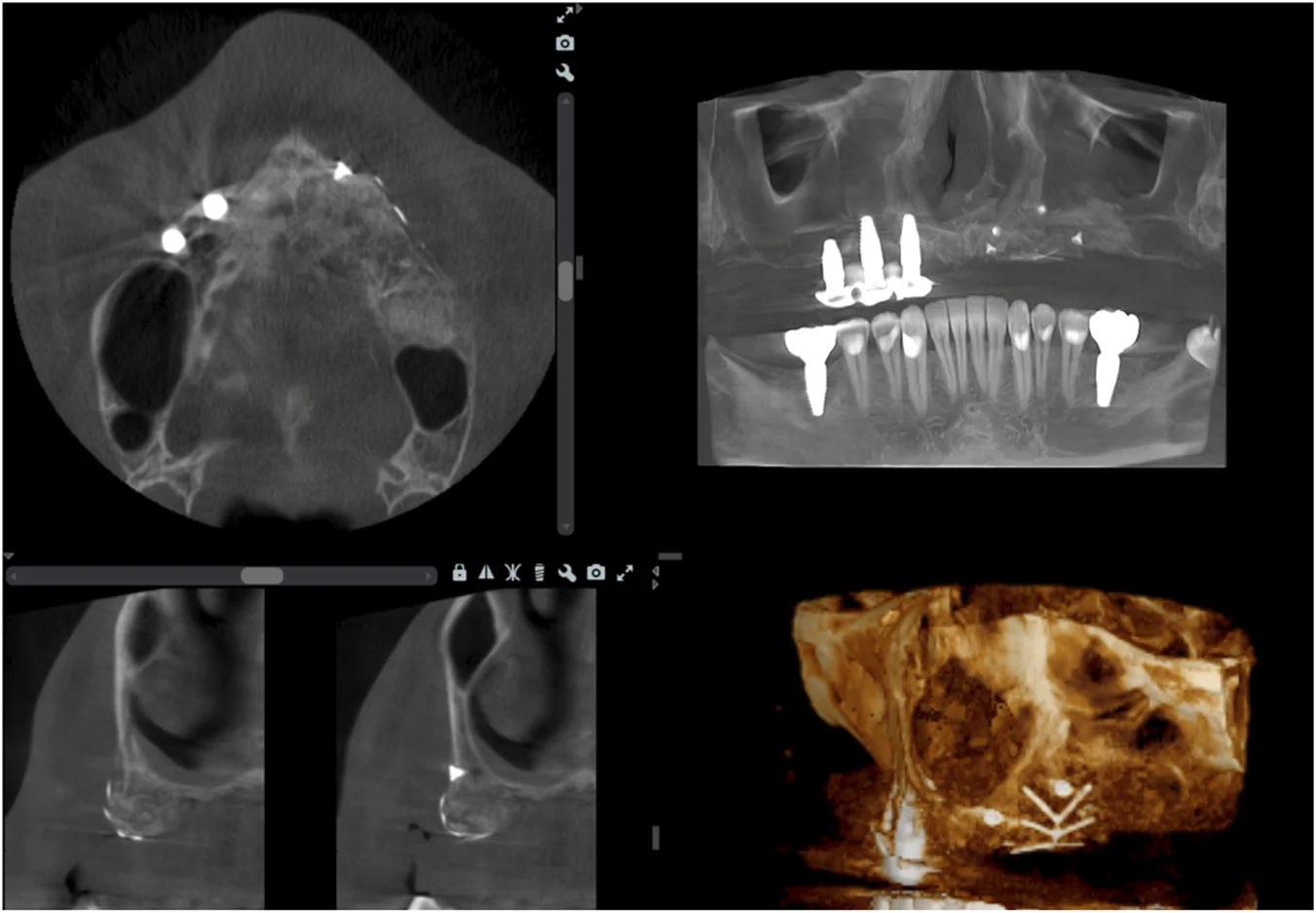
Case 2: 6 months post-grafting. CBCT image and reconstruction showing restoration of alveolar ridge height and width at 6 months post-grafting. Top left: CBCT axial view. Bottom left: CBCT coronal view. Top right: panoramic X-ray. Bottom right: 3D volumetric CBCT reconstruction.

Even though the patient had previously demonstrated poor wound healing with his previous surgeries, the grafted maxilla healed without complication. Excellent reconstruction of both vertical height and horizontal width was observed. Cross-sectional images and 3D imaging demonstrated that the desired alveolar ridge form was achieved (Figure 11).

**FIGURE 12:**
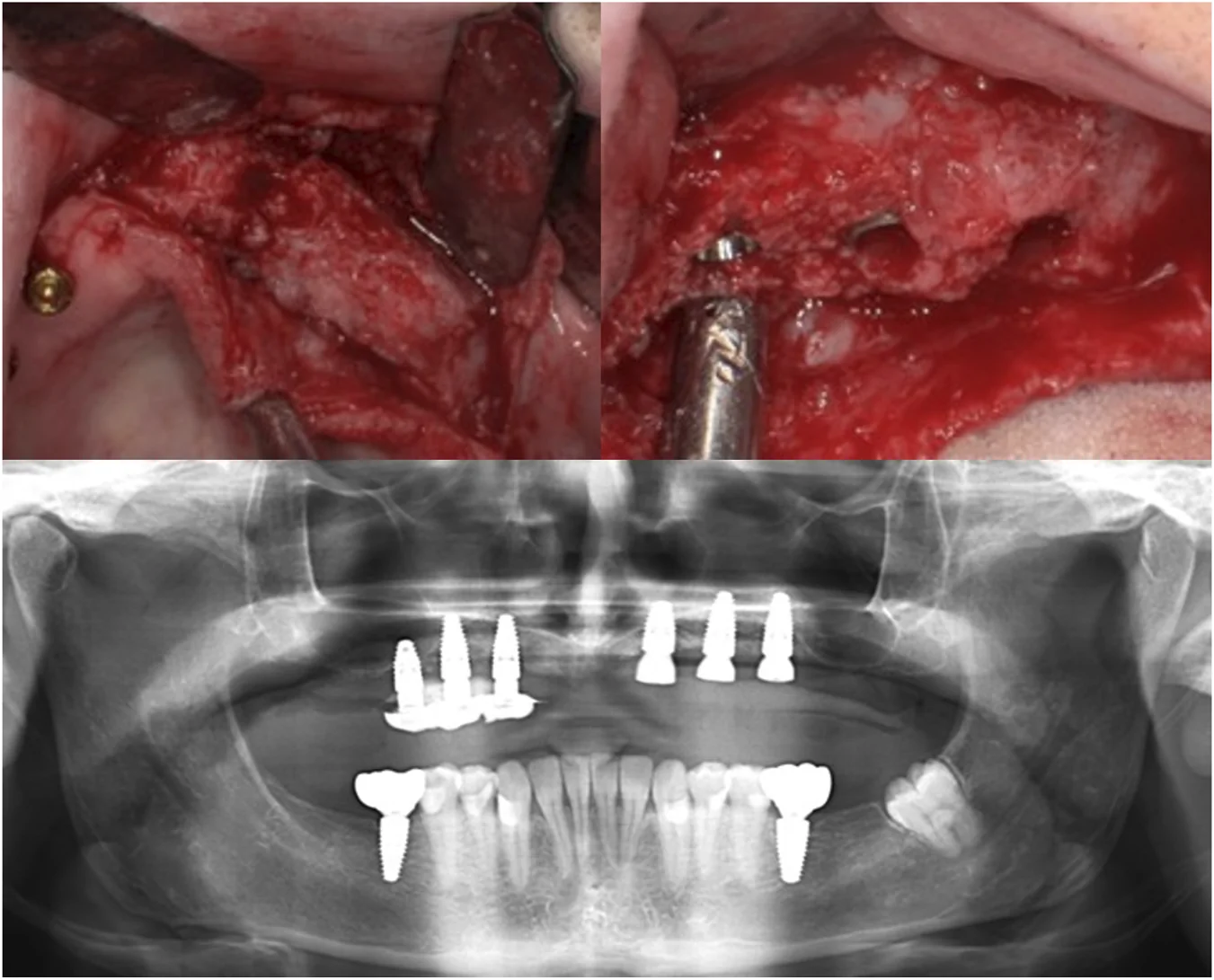
Case 2: implant placement and 6 months post-implant placement. Clinical photographs (top right and left) showing the quality of the bone at the time of dental implant placement. Panoramic radiograph (bottom) showing osteointegration of the three dental implants 6 months post placement.

At 8 months post ridge augmentation, the patient had the alveolus exposed and three dental implants (4.1 mm D × 10 mm L) placed into the regenerated alveolar ridge. The grafted bone was found to be Type II in density at the time of dental implant insertion (Figure 12).

The radiographic image at 6 months post dental implant placement demonstrated integration of the three implant fixtures (Figure 12).

## 4 Discussion

Bone grafts are considered to act via a combination of osteoconduction, where they act as a scaffold to support osteogenic tissue ingrowth, osteogenesis, where they provide osteogenic cells to form new bone, osteoinduction, where they provide osteoinductive signals (BMPs) that stimulate the host mesenchymal cells (MSCs) to differentiate into osteoblasts, and osteopromotion, where the graft provides one or more additional signals that enhance bone repair (Albrektsson and Johansson, 2001; Abjornson et al., 2014).

Natural Matrix Protein (NMP) is an allograft-derived bone graft that claims to have increased osteoregenerative potential due to increased growth factor bioavailability (Smith et al., 2026). Our results show that, on average, 40% more BMP-7 was released from NMP than from DBM produced from the same donor over 14 days, supporting the claim that the NMP process increases the bioavailability of BMP. Smith et al. (2026) proposed that the NMP process results in the redistribution of BMP from a tightly bound matrix compartment to a loosely bound compartment that is believed to reflect the incorporation of the growth factors into a complex of non-collagenous proteins. We also showed increased amounts of BMP-2, BMP-7, TGF-ß1, PDGF-bb, and VEGF complexed with NCPs in NMP compared to DBM in two donors (Supplementary Figure S1), suggesting that the NMP process affects other growth factors in addition to BMP-7.

The muscle pouch model allows for the evaluation of the entire process of bone formation, which involves the interaction of many different cells and tissues. Even when using athymic rats, which are T-cell deficient and do not produce a cytotoxic response to xenogenic materials, implantation of human tissue results in the recruitment of macrophages, giant cells, and granulocytes (ASTM, 2013; Rolstad, 2001). The muscle pouch model has been described as the “best method” of determining whether a material is osteoinductive (Albrektsson and Johansson, 2001) as any bone formed comes from induction of the MSCs present in the muscle. In contrast, if the osteoinductive agent were implanted in a bony site, it would not be possible to determine whether any new bone formed from osteoinduction of MSCs or due to the activity of osteoblasts present at the implant site. Several studies have evaluated commercially manufactured lots of DBM using this model (Edwards et al., 1998; Traianedes et al., 2004; Schwartz et al., 2011; Schwartz et al., 1998) and the American Society for Testing and Materials (ASTM) has developed a standard method, ASTM F2529-13, for performing the muscle pouch assay with the purpose that it be used to validate processes produce osteoinductive grafts, for lot release testing of DBM and to support osteoinductivity claims in regulatory premarket submissions (ASTM, 2013).

While histological scoring is typically used to assess bone formation in this model, this is laborious, can be subjective, and as only one to three sections are assessed, it is only semi-quantitative. Kawai and Urist (1988) demonstrated that the amount of induced bone can be quantitated radiologically, and several studies have used microCT to quantitate the amount of mineralized tissue formed following implantation of osteoinductive materials into a muscle pouch (Smith et al., 2026; Barr et al., 2010). This potentially allows for the standardization of the analysis of the recovered implants and also for the assessment of the entire volume, rather than only a small part of the implant.

To confirm the potential of using microCT to quantify bone formed in the muscle pouch, we investigated the correlation between the induced bone volume fraction (%MIN) measured by microCT and the % new bone measured histologically by preparing a series of test articles with different ratios of NMP to inactive DBM. The results showed that these two measures were very highly correlated (R^2^ = 0.97), supporting the use of microCT to quantitate bone formation in this model. While a dose response was noted at lower percentages of NMP, there was no difference in the %MIN or %NB between the 100% and 75% NMP groups. This maximal response likely reflects that the residual matrix occupies space and therefore limits the volume of new bone that can form within the implant volume.

MicroCT analysis of the unimplanted OsteoAMP, i-Factor, and the two cellular bone allografts (ViviGen and Trinity) showed that they all contained mineralized tissue prior to implantation. In contrast, the unimplanted NMP was radiolucent, allowing for identification of newly formed mineralized tissue at 28 days. While unimplanted INFUSE was not assessed by microCT, it comprises an acellular collagen sponge produced from bovine tendon collagen, which is soaked with a solution of rhBMP-2 and therefore does not contain any mineral prior to implantation (US Food and Drug Administration, 2004). The presence of pre-existing mineral made it difficult to quantify newly formed bone based on the microCT results. While we attempted to correct for the pre-existing mineral by subtracting the Unimplanted-%MIN from the Implanted-%MIN, this still resulted in a positive corrected %MIN for OsteoAMP and OsteoFactor + OsteoAMP, even though histologically they did not contain any new bone. Consequently, microCT should only be used when the initial implant does not contain mineral. The %MIN for the two lots of NMP were 17.2 ± 9.5% and 30.3 ± 8.1%, and the %MIN for INFUSE was 30.8 ± 10.2%. One possible explanation of the difference between the 2 NMP lots is donor variability in BMP content (Pietrzak et al., 2006), as the amount of bone formed is known to correlate to the amount of BMP present in the matrix (Murray et al., 2007). Histologically, both lots of NMP formed new bone in 100% of the recovered implants.

OsteoAMP is derived from bone allograft that has been processed with the bone marrow to maintain the growth factors present in native bone (Tidwell et al., 2017). Histologically, OsteoAMP did not form any new bone in the rat athymic muscle pouch. Furthermore, the addition of the allogeneic bone protein extract OsteoFactor to OsteoAMP did not result in any bone formation. This is surprising, as both OsteoAMP and OsteoFactor are reported to have high levels of BMPs (Tidwell et al., 2017; Go et al., 2014; OsteoFactor® Allogeneic Proteins FM, 2021). One possible explanation for the failure of these test articles to induce bone formation is that the BMP present within these test articles was inactivated during tissue processing or sterilization.

While several retrospective clinical studies have reported OsteoAMP resulted in high rates of fusion when used in spine surgery, these were primarily assessed by X-ray, and stated that “any radiodensity that obliterates or blurs the lucency between endplates and plugs that was observed on the postoperative films was considered an evidence of fusion” (Roh et al., 2013; Field, 2013; Yeung et al., 2014; Hani et al., 2024). As we observed that OsteoAMP contains mineralized bone prior to implantation, it is likely that this resulted in an overestimation of fusion in these studies. A recent case report concerning a 21-year-old who had undergone alveolar grafting with OsteoAMP assessed a tissue biopsy recovered 5 months post grafting. While CT scans at 3 months showed the presence of mineralized tissue at the graft site, histologically the excised tissue comprised non-vital bone surrounded by vascularized fibrous tissue. Immunostaining failed to identify any osteoblasts or osteoclasts (Grisham et al., 2022). Consequently, we conclude that OsteoAMP should primarily be considered to act as an osteoconductive scaffold.

i-Factor comprises P-15 peptide absorbed onto granules of xenogenic bone that have been heated to remove all organic material, resulting in an anorganic bone matrix (ABM) comprised of dense hydroxyapatite. The P-15 peptide mimics the cell-binding domain of Type I collagen. This peptide is reported to attract, attach, and activate osteogenic cells, encouraging them to proliferate and differentiate at the graft site (Nguyen et al., 2003). While radiologically mineralized tissue was visible in the recovered implants, it was determined histologically that the recovered tissue comprised ABM granules surrounded by a dense inflammatory tissue with no new bone visible in any of the implants. Two studies have recently evaluated i-Factor in a sheep spine fusion model and reported that while mineralized tissue was visible throughout the fusion mass, histologically new bone formation was only observed adjacent to the host bone surface. In contrast, bone formation at the core of the fusion mass was absent, demonstrating that i-Factor supported ingrowth from the local bone but was not osteoinductive or osteogenic (Axelsen et al., 2019; Kucko et al., 2025). Therefore, i-Factor should primarily be considered an osteoconductive scaffold.

The cellular bone allografts (CBAs) evaluated comprise cancellous bone, with viable cells combined with demineralized bone fibers. Histologically, new bone was observed in 7 of 10 of the ViviGen implants and 10 of 10 of the Trinity implants. While these implants showed a degree of bone-forming activity, they comprise both cells and DBM, which may possess osteoinductive activity. A study comparing the efficacy of CBA with living cells to CBA with cells killed by freeze-thawing, and to DBM in a rat spine fusion model showed that the presence of viable cells did not result in any additional benefit and concluded that “the DBM component is probably the key determinant of fusion” (Abedi et al., 2020).

The INFUSE bone graft comprises a collagen sponge that is incubated with a solution of rhBMP-2. INFUSE consistently formed new bone in this model. However, this bone formed as cyst-like ossicles comprising a thin shell of mineralized bone surrounding a non-mineralized core. The size of these ossicles was significantly smaller than any of the other recovered implants. Similar results have been observed previously when INFUSE is evaluated in this model. It was concluded that the cyst formation is due to the supra-physiologic dose of BMP applied (1.5 mg/mL) and the burst release of BMP from the sponge over the first 24 h (Smith et al., 2026). The total volume of the INFUSE implant was less than 50% of the initial volume of BMP solution added to each implant (29 ± 13 mm^3^ vs. 70 mm^3^). This result suggests that while INFUSE kits are sized based on the volume of BMP solution (i.e., the XXS kit is 0.7 mL), this should not be relied upon as an estimate of the volume that will be restored.

NMP implants comprised a mixture of connective tissue and ossicles of new bone formed onto the residual NMP matrix surrounding marrow. No signs of inflammation or edema were observed. Histologically, 10 of 10 implants contained new bone.

The two clinical cases described successful regeneration of alveolar bone at the graft site and the successful placement of dental implants. Clinically, healing in both patients was uneventful, and there were no reports of excessive swelling or inflammation, which has been reported when using rhBMP-2 grafts (Zara et al., 2011; Woo, 2013). Both cases represent challenging environments for successful regeneration where simple osteoconductive materials would not be expected to result in successful outcomes. A recent study reported on the use of NMP in one- and two-level lumbar spine fusions in 50 patients, 98% of whom had at least one comorbidity and 72% had two or more (including diabetes, current smokers, and extreme obesity). Of those 50 patients, 100% were assessed as fused based on X-ray assessment of the lack of motion of the vertebrae and no signs of radiolucency, and 89% showed complete bony bridging on CT, with the remaining 11% showing bone within the interbody space (Nunley et al., 2025). Based on the results of the current study, NMP appears to be regenerative rather than osteoconductive and is able to promote bone regeneration in patients with comorbidities.

A limitation of this study is that we only assessed a single lot, or in the case of NMP, two lots, of the various allograft-based test articles. It is known that there is significant donor-to-donor variability, which could result in the different outcomes (Murray et al., 2007; Bae et al., 2010). While all test articles used in this study were from commercial lots and so reflect what is currently available to clinicians, future studies should be performed evaluating a large number (25+) of lots to definitively assess the lot-to-lot variability for each product. Similarly, the clinical study is limited to two cases without any case controls.

## 5 Conclusion

Assessment of bone formation based on radiological imaging must consider the potential confounding effects of any mineralized material present in bone graft materials at the time of implantation.

i-Factor and OsteoAMP, on its own or supplemented with OsteoFactor, did not show any osteoregenerative potential in this study. The ViviGen showed variable osteoregenerative potential, while Trinity, INFUSE, and NMP were consistently osteoregenerative in the athymic rat muscle pouch model. NMP was observed to support bone regeneration clinically.

## Data Availability

The raw data supporting the conclusions of this article will be made available by the authors, without undue reservation.
