# Supplementary material for: A novel growth factor bioavailability-enhanced allograft for use in craniomaxillofacial bone regeneration
Source: Front Bioeng Biotechnol. 2026 Sep 8;14:1841387. doi: 10.3389/fbioe.2026.1841387 (PMC13595062; doi:10.3389/fbioe.2026.1841387)
Supplement: Supplementary file 1 [file Table1.docx]

A novel growth factor bioavailability enhanced allograft (GFBA) for use in craniomaxillofacial bone regeneration

Sean A.F. Peel^1,2*^, Jeff Terryberry1, Shiva Naseri^1^, Charles F. Orth^3,4^

Supplemental Data

***Supplemental Tables***

*Table S1:* Manufacturer of Test Articles

| **Test Article** | **Manufacturer** | **Description** |
| --- | --- | --- |
| OSTEOAMP | BioVentus Surgical | Processed bone allograft with marrow |
| OsteoFactor | XTANT Medical | cocktail of allogenic growth factors, stabilized by native human collagen |
| i-Factor Putty | Cerapedics | P15 peptide on anorganic bone matrix (xenograft) suspended in a hydrogel carrier |
| ViviGen Formable Cellular Bone Matrix | LifeNet | cellular bone allograft (lineage committed cells) |
| Trinity Elite | Orthofix | Cellular bone allograft (mixed cells) |
| INFUSE Bone Graft | Medtronic | rhBMP-2 on a collagen sponge |
| NMP | Induce Biologics USA | GFBA |

Figure Legends

*Figure S1:* Growth factors extracted from DBM and NMP from 2 human donors

DBM and NMP was produced from 2 donors as described in the materials and methods. A 250 mg aliquot of each lyophilized test article was extracted with 10mL of 50mM AcOH for 24 hours at room temperature on a rocker to extract the growth factors associated with the BMP/NCP complex. [1] The supernatant was collected, aliquoted and stored at -20°C until it was assayed for BMP-2, BMP-7, TGF-ß1, PDGF-bb or VEGF using the respective Quantikine ELISA’s (R&D Systems, Minneapolis, MN)

Reference

1.	Smith, M.-S.R., et al., *Development of a growth factor bioavailability enhanced allograft (GFBA) for bone regeneration.* Cell and Tissue Banking, 2026. **27**: p. 6.

*Figure S2*: H&E stained sections of various test article recovered 28 days after placement in an athymic rat muscle pouch. Original Magnification 2x. Scale bar 2mm, Field of view 8.5cm

OsteoAMP (A), OsteoFactor + OsteoAMP (B), i-Factor (C), ViviGen (D), Trinity (E), INFUSE (F), NMP (G)
